# Radiocarbon dating of dental tissues for determining time since death in forensic cases: a systematic review

**DOI:** 10.1007/s00414-025-03666-0

**Published:** 2025-11-24

**Authors:** Chantal Milani, Massimo Lancia, Cristiana Gambelunghe, Niccolò Pini, Lucio Calcagnile, Gianluca Quarta, Marisa D’Elia, Roberto Scendoni, Piergiorgio Fedeli, Luca Tomassini

**Affiliations:** 1 Unit of Forensic Pathology, Department of Medicine, Surgery and Dentistry “Schola Medica Salernitana”, University of Salerno, Baronissi, 84081 Italy; 2 Forensic Medicine, Forensic Science and Sports Medicine Section, Department of Medicine and Surgery, University of Perugia, Piazza Lucio Severi, Perugia, 06132 Italy; 3 CEDAD (Centre for Applied Physics, Dating and Diagnostics), Department of Mathematics and Physics “Ennio de Giorgi”, University of Salento, Lecce, 73100 Italy; 4 Department of Law, Institute of Legal Medicine, University of Macerata, Macerata, 62100 Italy; 5 School of Law, Legal Medicine, University of Camerino, Camerino, 62032 Italy; 6 International School of Advanced Studies, University of Camerino, Camerino, 62032 Italy

**Keywords:** Radiocarbon dating, Dental tissues, Forensic anthropology, Postmortem changes

## Abstract

**Objective:**

Radiocarbon analysis is used in forensic anthropology, and its application to dental tissues has become increasingly adopted. This systematic review aimed to assess the current state of knowledge on radiocarbon dating of dental tissues, focusing on methodological approaches, interpretative frameworks, and forensic applicability of enamel, dentin, and cementum.

**Methods:**

PubMed, Scopus, and Web of Science databases were searched up to March 2024, and original studies involving radiocarbon analysis of dental tissues for forensic or anthropological purposes were included. Nine studies were found eligible and reviewed in detail.

**Results:**

The selected studies encompassed over 270 individual teeth, primarily molars, and were conducted using accelerator mass spectrometry combined with calibration curves. Enamel yielded the most accurate results, with a mean absolute error of ± 1.3–1.9 years for post-1963 formation. Dentin and cementum, although metabolically active and subject to appositional changes, contributed useful complementary information but introduced potential chronological discrepancies. Key limitations included small sample sizes, practical constraints in separating dental tissues, and the impact of geographic and dietary variability on radiocarbon interpretation.

**Conclusions:**

Radiocarbon dating of dental tissues, particularly enamel, offers a reliable means of estimating time-related biological parameters in forensics. Nonetheless, its effectiveness is influenced by sample integrity, the specific dental component analyzed, and the availability of contextual data, such as geographic origin. Integration with stable isotopes and genetic analyses is recommended to enhance the robustness of forensic identification. Future studies should prioritize larger sample sizes and focus on whole-tooth analyses to reflect real-world forensic constraints.

## Introduction

Radiocarbon analysis, especially in the form of bomb pulse dating, plays a crucial role in modern forensic science for estimating the time since death [1–3]. By measuring carbon-14 (C-14) levels in human tissues, this method can provide an approximate range of time since death. Although initially developed and widely used in archaeology to date ancient remains, its application has expanded to forensics, where it helps distinguish between historical and contemporary cases and supports accurate postmortem interval (PMI) estimations [4, 5].

This methodological shift has been particularly significant in the study of hard tissues, such as teeth, which have long played a pivotal role in many assessments in forensic investigations, identification, and anthropological research. Their resistance to postmortem decay and well-documented developmental timeline make them uniquely valuable for age estimation and contribution to the development of anthropological profiles or biological histories [6–11]. Among the various tools used for such purposes, radiocarbon dating—particularly through analysis of (C-14)—has become increasingly important, especially with the advent of the “bomb pulse” method [3, 12].

This approach takes advantage of the considerable increase in atmospheric C-14 levels caused by nuclear weapons testing during the mid-20th century [12–14]. Consequently, it is possible to align the C-14 concentration found in dental tissues, such as enamel or dentin, with specific points on the atmospheric C-14 curve. This technique enables accurate estimations of birth year or, in some cases, time of death for individuals who formed these tissues after the 1950 s [15].

However, teeth are complex anatomical structures comprising multiple tissues with distinct metabolic characteristics. For instance, enamel is formed during early life and is metabolically stable thereafter, whereas dentin and cementum may undergo limited post-formational changes [6]. This variability implies that the choice of dental component to be analyzed and how its radiocarbon signal is interpreted can significantly affect the estimated date [16]. Several studies have highlighted that separately analyzing enamel and dentin from the same tooth can provide different chronological windows, allowing for refined estimations of both birth and death years [17].

A key advantage of using teeth in radiocarbon analysis is their biological stability. Unlike bones, which are subject to remodeling and turnover throughout life, enamel retains the isotopic signal present at the time of its formation. This makes teeth particularly suitable for 14 C-based dating, especially in contexts where skeletal remains are incomplete or compromised by environmental conditions [18].

Radiocarbon dating of teeth has recently gained traction in forensic settings involving unidentified individuals, such as undocumented migrants or disaster victims, where precise age estimation is both a scientific and humanitarian priority. Despite its promise, literature on C-14 analysis of dental tissues remains sparse. Moreover, no clear consensus has been reached regarding the optimal methodology, the comparative reliability of enamel versus dentin, or the realistic margins of error for age estimation across different populations.

Furthermore, individual factors, such as sex, diet, and geographic origin, can subtly influence C-14 levels and must be carefully considered during interpretation. For example, Thevissen et al. [6] recently reported that environmental and nutritional parity could help eliminate confounding variables, enabling more reliable population comparisons in tooth development studies.

Given these gaps and challenges, this review was conducted to systematically assess the current state of knowledge on radiocarbon analysis in dental tissues. We aimed to examine the methodologies employed, the specific dental components analyzed, and the forensic relevance and interpretation issues of the resulting data.

## Materials and methods

### Study design and search strategy

This systematic review was conducted in accordance with the Preferred Reporting Items for Systematic Reviews and Meta-Analyses (PRISMA) guidelines to ensure transparency and methodological rigor throughout the process. Although this work has forensic implications, it was not registered in PROSPERO because the registry does not encompass reviews unrelated to health or social care.

A systematic search was performed across three major electronic databases—PubMed, Web of Science, and Scopus—from their inception to March 2024. The search strategy was derived from a previously validated string used for radiocarbon research, specifically focusing on dental tissues and bomb-pulse C-14 dating. The following MeSH terms and keyword combinations were used.

((“Time Factors“[MeSH] OR time) AND (“Death“[MeSH] OR deaths OR “Postmortem Changes“[MeSH] OR postmortem OR “post-mortem”) AND (interval OR intervals) AND (“Radiocarbon Dating” [2][MeSH] OR “Carbon-14“[MeSH] OR radiocarbon OR “C14” OR “carbon dating” OR “radiometric dating” OR radiometric) AND (“Anthropology, Forensic“[MeSH] OR “forensic anthropology” OR (forensic AND anthropology)) AND (“Tooth“[MeSH] OR teeth OR tooth) AND (“bomb peak” OR “bomb curve” OR bomb OR bombs))

Language or date restrictions were not applied. Additional studies were identified by manually screening the references of all selected articles.

### Eligibility criteria

The inclusion criteria for this review were as follows:

1. Original studies involving C-14 analysis of dental tissues (enamel, dentin, or cementum).
2. Articles focused on forensic or anthropological applications.
3. Studies evaluating the correlation between C-14 levels and estimated time of birth or death.
4. Full texts available in English.

The exclusion parameters were as follows:

1. Studies focused only on bones or soft tissues.
2. Archaeological contexts not aimed at recent or forensic relevance.
3. Reviews, conference abstracts, commentaries, or editorials.
4. Studies that did not provide sufficient methodological details (e.g., no data on sample preparation or interpretation model).
5. Papers including dental samples only for calibration purposes or without specific reference to bomb pulse methodology.

### Study selection

Title, abstract, and full-text screening were independently carried out by three researchers using Rayyan AI software [19, 20]. The reference lists of all identified sources were thoroughly reviewed and cross-checked to identify additional relevant literature. Each document underwent a detailed methodological assessment in line with PRISMA guidelines, including a careful evaluation of potential bias.

### Data extraction and categorization

The data collection process involved both the selection of studies and the extraction of relevant data. All papers with potentially relevant titles or abstracts were independently reviewed by the researchers. Disagreements regarding study eligibility were resolved through discussion and consensus. Initial data extraction was performed by three investigators and subsequently verified by a fourth investigator.

Since the aim of this review was to provide tools to identify the most accurate PMI, studies that met this criterion were selected and described in Table 1, including C-14 data from samples of forensic interest, in which the anatomical region, lag time, age, and sex were specified.

**Table 1:** Summary of the main studies on radiocarbon dating of dental tissues included in the systematic review. For each study, information on sample characteristics (number of individuals, age, sex, and type of tooth analyzed), target (year of birth, year of death), results, and estimated lag time are reported

Authors	Year of pub	individuals	age	sex	*N*. samples	Details	Target	Dating	Results	Lagtime
Alkass et al. [15]	2011	84	N/A	47 M; 8 NK; 29 F	95	Molar, premolar	YOB	1955–1963 (12) powt 1963 (66) pre bomb peak (17)	The study analyzed 95 teeth from 84 individuals, categorizing samples into three groups based on enamel formation timing relative to the bomb pulse. The average error in age estimation ranged between 1.3 and 1.9 years, with excellent correlation for teeth formed after 1963 (R² = 0.989). ¹³C analysis revealed significant differences among individuals from various geographical regions, proving useful for forensic identification. The combination of ¹³C and ¹⁴C in dental enamel remains a reliable method for determining age and geographical origin, with valuable forensic and investigative applications.	1,3 − 1,9 yr
Bertrand et al. [21]	2024	1	48–58 yrs	F	1 skeleton	Upper and lower canines	YOB	lower canine (1970–1972); upper canine (1972–1974 or 1962 − 1932)	Radiocarbon dating, including bomb-pulse analysis, proves crucial for determining the archaeological or forensic significance of human remains and refining the PMI. Case studies demonstrate its effectiveness in identifying birth and death dates through various biological tissues. Despite challenges like lag time and tissue-specific factors, radiocarbon dating remains a reliable and cost-effective tool, offering precise PMI estimates in a short time. Future research should focus on variations across tissues, age groups, and the decreasing radiocarbon concentration in recent years to refine post-2010 forensic dating.	8 yrs
Handlos et al. [12]	2018	2	55–56 yrs	M	12 (6 + 6)	N/A	N/A	Post 1950	This pilot study assessed the use of radiocarbon (14 C) dating to determine the time of death from skeletal remains. It found that samples with short carbon turnover times, such as hair, nails, and bone fat, provided more accurate estimates of the time of death, while bone collagen, with its longer and variable turnover, was less precise. The study also highlighted the potential of dental samples to estimate the age at death. Overall, the findings suggest that using a combination of different sample types can improve the reliability of time-of-death estimates, though further research is needed to refine these methods.	N/A
Ubelaker et al. [22]	2006	2	One is 70 yrs; one is 33 yrs	F	2	left mandibular canine; left lateral incisor	YOB, YOD	1925–1995	The radiocarbon analysis of eight samples from two individuals showed that dental samples predate the bomb curve, indicating formation before 1950. The bone samples generally contained bomb carbon, except for one cortical bone sample, which showed minimal post − 1954 carbon, suggesting a delay in incorporating new carbon. For the individual born in 1925, the trabecular bone formed around 1954, just before death, while the cortical bone’s formation was not dated as closely. The second individual, who lived until 1995, had both bone types with bomb carbon values, indicating formation around 1956–1957. These results reflect the average radiocarbon content of the bone collagen and suggest limited new amino acid incorporation in later years.	5 y in F33 and 38–39 y
Calcagnile et al. [13]	2013	1	36 yrs	M	4	right mandibular canine; 2nd right mandibular molar	YOB, YOD	YOB 1973	Bone collagen showed different lag times due to varying turnover rates: 18.1 years for cortical bone (S4) and 4.9 years for trabecular bone (S5). Tooth enamel analysis estimated a birth year of 1972.0 ± 1.8, consistent with the known 1973 birth date.	1,8 yrs
Wang et al. [23]	2010	13	N/A	5 M; 3 F; 5NK	13	first molar; second molar; third molar	YOB	YOB (1953–2000)	The study analyzed 13 teeth from individuals born between 1953 and 2000 using	1,9 yrs
Alkass et al. [18]	2013	33	N/A	22 M; 11 F	66	incisor (central and lateral), canines, premolars and molars (first, second and third)	YOB	YOB(1900–2000)	The study analyzed 66 teeth from 33 individuals to estimate their year of birth, geographical origin, and sex. Radiocarbon (14 C) analysis provided birth year estimates with an average error of ± 1.8 years. Stable isotopes (13 C and 18O) helped determine geographical origin, while DNA analysis confirmed sex and individual profiles. These methods improve forensic identification by narrowing down possible matches.	1–2 yrs
Douglas et al. [5]	2011	4	16y, 27y, 44y, 56y	N/A	4	dental enamel of permanent molar	YOB; YOD	**Individual A**: born in 1947, died in 1991 **Individual B**: born in 1941, died in 1997 **Individual C**: born in 1964, died in 1991 **Individual D**: born in 1968, died in 1984	The study analyzed four individuals using radiocarbon (14 C) analysis of dental enamel and bones. Enamel provided accurate birth year estimates, while bone (cortical and trabecular) helped determine the death year, considering bone remodeling rates. The method proved effective for forensic identification​	0
Cook et al. [24]	2006	8	N/A	N/A	8	molars (third molars or wisdom teeth), incisors, premolars	YOB	YOB(1953–2000)	The study analyzed eight teeth to estimate the year of birth using radiocarbon (14 C) analysis. Enamel provided two possible birth year ranges, and dentine/cementum helped select the correct one. The method proved accurate for forensic identification​	0

The extracted data were categorized based on the analyzed dental tissue (enamel, dentin, or cementum), when such specification was available. This classification enabled the evaluation of the influence of tissue-specific turnover and appositional dynamics on the resulting C-14 measurements, as described in Table 2. Although the data in Table 2 included sex, this variable was not considered in the analysis because further data subdivision would have reduced the already small sample size, compromising any statistical approach.

**Table 2:** Dataset of individual dental samples included in the systematic review. The table reports information on sample ID, location, age, sex, tooth type, estimated formation period, year of birth or death (when available), error, lag time and compensation, scope (forensic or research), and dental section analyzed

Source	Sample ID	Location	Age	Sex	Tooth *n*.	tooth formation time	DOB	estimated age	error	Lag Time	lag time compensation	Death	scope	archeological	Dental section
Analysis of 14 C and 13 C in teeth provides precise birth dating and clues to geographical origin	FM01	Sweden	N/A	M	44	5,1	1991,3	1989,9	−1,4	N/A	N/A	N/A	research	No	coronal enamel
Analysis of 14 C and 13 C in teeth provides precise birth dating and clues to geographical origin	FM02	Sweden	N/A	M	34	5,2	1991,8	1990,8	−1	N/A	N/A	N/A	research	No	coronal enamel
Analysis of 14 C and 13 C in teeth provides precise birth dating and clues to geographical origin	FM03	Sweden	N/A	M	44	4,2	1993,2	1993,5	−0,4	N/A	N/A	N/A	research	No	coronal enamel
Analysis of 14 C and 13 C in teeth provides precise birth dating and clues to geographical origin	FM05	Sweden	N/A	F	44	4,2	1988,8	1988	−0,8	N/A	N/A	N/A	research	No	coronal enamel
Analysis of 14 C and 13 C in teeth provides precise birth dating and clues to geographical origin	FM06	Sweden	N/A	F	14	4,9	1988,8	1985,7	−3,1	N/A	N/A	N/A	research	No	coronal enamel
Analysis of 14 C and 13 C in teeth provides precise birth dating and clues to geographical origin	FM07	N/A	N/A	M	34	5,1	1988,6	1987,6	−1	N/A	N/A	N/A	research	No	coronal enamel
Analysis of 14 C and 13 C in teeth provides precise birth dating and clues to geographical origin	FM08	N/A	N/A	F	44	4,4	1992,5	1991,4	−1,1	N/A	N/A	N/A	research	No	coronal enamel
Analysis of 14 C and 13 C in teeth provides precise birth dating and clues to geographical origin	FM09	N/A	N/A	F	24	4,9	1990,5	1990	−0,5	N/A	N/A	N/A	research	No	coronal enamel
Analysis of 14 C and 13 C in teeth provides precise birth dating and clues to geographical origin	FM10	Sweden	N/A	M	21	3,2	1982	1981,2	−1,3	N/A	N/A	N/A	research	No	coronal enamel
Analysis of 14 C and 13 C in teeth provides precise birth dating and clues to geographical origin	FM11	Sweden	N/A	F	35	5,7	1989,2	1989,2	0	N/A	N/A	N/A	research	No	coronal enamel
Analysis of 14 C and 13 C in teeth provides precise birth dating and clues to geographical origin	FM12	N/A	N/A	F	14	4,9	1991,3	1992,3	−1	N/A	N/A	N/A	research	No	coronal enamel
Analysis of 14 C and 13 C in teeth provides precise birth dating and clues to geographical origin	FM25	Sweden	N/A	F	34	4,4	1993,6	1994	−0,4	N/A	N/A	N/A	research	No	coronal enamel
Analysis of 14 C and 13 C in teeth provides precise birth dating and clues to geographical origin	FM27	N/A	N/A	M	14	5,6	1990,8	1989,7	−1,1	N/A	N/A	N/A	research	No	coronal enamel
Analysis of 14 C and 13 C in teeth provides precise birth dating and clues to geographical origin	FM29	Sweden	N/A	F	24	4,9	1991,8	1991,3	−0,5	N/A	N/A	N/A	research	No	coronal enamel
Analysis of 14 C and 13 C in teeth provides precise birth dating and clues to geographical origin	FM30	Sweden	N/A	F	14	4,9	1989,1	1987,3	−1,8	N/A	N/A	N/A	research	No	coronal enamel
Analysis of 14 C and 13 C in teeth provides precise birth dating and clues to geographical origin	FM31	Sweden	N/A	F	17	5,8	1993,4	1992,3	−1,1	N/A	N/A	N/A	research	No	coronal enamel
Analysis of 14 C and 13 C in teeth provides precise birth dating and clues to geographical origin	FM32	Sweden	N/A	F	14	4,9	1991,1	1990,3	−0,8	N/A	N/A	N/A	research	No	coronal enamel
Analysis of 14 C and 13 C in teeth provides precise birth dating and clues to geographical origin	FM33	Sweden	N/A	F	14	4,9	1991,5	1990,6	−0,9	N/A	N/A	N/A	research	No	coronal enamel
Analysis of 14 C and 13 C in teeth provides precise birth dating and clues to geographical origin	FM34	Sweden	N/A	F	34	4,4	1991,4	1990,8	−0,6	N/A	N/A	N/A	research	No	coronal enamel
Analysis of 14 C and 13 C in teeth provides precise birth dating and clues to geographical origin	FM39	N/A	N/A	M	34	5,1	1990,8	1989,9	−0,9	N/A	N/A	N/A	research	No	coronal enamel
Analysis of 14 C and 13 C in teeth provides precise birth dating and clues to geographical origin	FM42	N/A	N/A	M	14	5,6	1993,2	1992,5	−0,7	N/A	N/A	N/A	research	No	coronal enamel
Analysis of 14 C and 13 C in teeth provides precise birth dating and clues to geographical origin	FM50	Sweden	N/A	F	14	4,9	1988,3	1987,1	−1,2	N/A	N/A	N/A	research	No	coronal enamel
Analysis of 14 C and 13 C in teeth provides precise birth dating and clues to geographical origin	FM53	N/A	N/A	M	14	5,6	1990,9	1989,3	−1,6	N/A	N/A	N/A	research	No	coronal enamel
Analysis of 14 C and 13 C in teeth provides precise birth dating and clues to geographical origin	FM85	N/A	N/A	F	47	5,6	1963,7	1963,4	−0,3	N/A	N/A	N/A	research	No	coronal enamel
Analysis of 14 C and 13 C in teeth provides precise birth dating and clues to geographical origin	OC29	N/A	N/A	M	38	13	1950,6	1944,8	−5,8	N/A	N/A	N/A	research	No	coronal enamel
Analysis of 14 C and 13 C in teeth provides precise birth dating and clues to geographical origin	OC41	N/A	N/A	F	45	5,7	1973,7	1972,7	−1	N/A	N/A	N/A	research	No	coronal enamel
Analysis of 14 C and 13 C in teeth provides precise birth dating and clues to geographical origin	OC42	N/A	N/A	F	18	11,2	1965,3	1968	−2,7	N/A	N/A	N/A	research	No	coronal enamel
Analysis of 14 C and 13 C in teeth provides precise birth dating and clues to geographical origin	OC46	N/A	N/A	M	48	13	1957,4	1956,3	−1,1	N/A	N/A	N/A	research	No	coronal enamel
Analysis of 14 C and 13 C in teeth provides precise birth dating and clues to geographical origin	OC47	N/A	N/A	F	28	11,2	1956,8	1955,2	−1,6	N/A	N/A	N/A	research	No	coronal enamel
Analysis of 14 C and 13 C in teeth provides precise birth dating and clues to geographical origin	OC48	N/A	N/A	F	38	11,8	1971,2	1970,6	−0,6	N/A	N/A	N/A	research	No	coronal enamel
Analysis of 14 C and 13 C in teeth provides precise birth dating and clues to geographical origin	OC51	N/A	N/A	F	28	11,2	1969,3	1968	−1,3	N/A	N/A	N/A	research	No	coronal enamel
Analysis of 14 C and 13 C in teeth provides precise birth dating and clues to geographical origin	OC52	N/A	N/A	M	28	12,6	1985,7	1983,5	−2,2	N/A	N/A	N/A	research	No	coronal enamel
Analysis of 14 C and 13 C in teeth provides precise birth dating and clues to geographical origin	0C59	N/A	N/A	M	18	12,6	1962,6	1961,1	−1,5	N/A	N/A	N/A	research	No	coronal enamel
Analysis of 14 C and 13 C in teeth provides precise birth dating and clues to geographical origin	OC61	N/A	N/A	F	28	11,2	1968,8	1968,4	−0,4	N/A	N/A	N/A	research	No	coronal enamel
Analysis of 14 C and 13 C in teeth provides precise birth dating and clues to geographical origin	OC62	N/A	N/A	M	28	12,6	1956,7	1956	−0,7	N/A	N/A	N/A	research	No	coronal enamel
Analysis of 14 C and 13 C in teeth provides precise birth dating and clues to geographical origin	OC65	N/A	N/A	M	48	13	1964,9	1963,5	−0,4	N/A	N/A	N/A	research	No	coronal enamel
Analysis of 14 C and 13 C in teeth provides precise birth dating and clues to geographical origin	OC67	N/A	N/A	M	18	12,6	1977,1	1981	−3,9	N/A	N/A	N/A	research	No	coronal enamel
Analysis of 14 C and 13 C in teeth provides precise birth dating and clues to geographical origin	OC69	N/A	N/A	M	37	6,5	1979,4	1977,4	−2	N/A	N/A	N/A	research	No	coronal enamel
Analysis of 14 C and 13 C in teeth provides precise birth dating and clues to geographical origin	OC71	N/A	N/A	M	27	6,5	1989,6	1990,5	−0,9	N/A	N/A	N/A	research	No	coronal enamel
Analysis of 14 C and 13 C in teeth provides precise birth dating and clues to geographical origin	OC72	N/A	N/A	F	28	11,2	1977,2	1976,8	−0,4	N/A	N/A	N/A	research	No	coronal enamel
Analysis of 14 C and 13 C in teeth provides precise birth dating and clues to geographical origin	OC73	N/A	N/A	F	15	5,6	1988,6	1988,2	−0,4	N/A	N/A	N/A	research	No	coronal enamel
Analysis of 14 C and 13 C in teeth provides precise birth dating and clues to geographical origin	OC80	N/A	N/A	F	48	11,8	1961,7	1959,1	−2,6	N/A	N/A	N/A	research	No	coronal enamel
Analysis of 14 C and 13 C in teeth provides precise birth dating and clues to geographical origin	J1	Japan	N/A	F	13	3,8	1967,3	1966,3	−1	N/A	N/A	N/A	research	No	coronal enamel
Analysis of 14 C and 13 C in teeth provides precise birth dating and clues to geographical origin	J2	Japan	N/A	M	14	5,6	1964,2	1962,8	−1,4	N/A	N/A	N/A	research	No	coronal enamel
Analysis of 14 C and 13 C in teeth provides precise birth dating and clues to geographical origin	FM41	N/A	N/A	M	28	12,6	1967,8	1965,6	−2	N/A	N/A	N/A	research	No	coronal enamel
Analysis of 14 C and 13 C in teeth provides precise birth dating and clues to geographical origin	FM41	N/A	N/A	M	36	2,4	1967,8	1968,2	−0,4	N/A	N/A	N/A	research	No	coronal enamel
Analysis of 14 C and 13 C in teeth provides precise birth dating and clues to geographical origin	FM41	N/A	N/A	M	26	3,3	1967,8	1967,6	−0,2	N/A	N/A	N/A	research	No	coronal enamel
Analysis of 14 C and 13 C in teeth provides precise birth dating and clues to geographical origin	FM41	N/A	N/A	M	14	5,6	1967,8	1966,5	−1,3	N/A	N/A	N/A	research	No	coronal enamel
Analysis of 14 C and 13 C in teeth provides precise birth dating and clues to geographical origin	FM41	N/A	N/A	M	15	6,6	1967,8	1965,5	−2,3	N/A	N/A	N/A	research	No	coronal enamel
Analysis of 14 C and 13 C in teeth provides precise birth dating and clues to geographical origin	FM57	Chile	N/A	F	38	11,8	1955,9	1957,8	−1,9	N/A	N/A	N/A	research	No	coronal enamel
Analysis of 14 C and 13 C in teeth provides precise birth dating and clues to geographical origin	U2-35	Uruguay	N/A	M	35	6,5	1966,8	1965,8	−1	N/A	N/A	N/A	research	No	coronal enamel
Analysis of 14 C and 13 C in teeth provides precise birth dating and clues to geographical origin	U3-25	Uruguay	N/A	M	25	6,6	1977,4	1976,8	−0,6	N/A	N/A	N/A	research	No	coronal enamel
Analysis of 14 C and 13 C in teeth provides precise birth dating and clues to geographical origin	U6-28	Uruguay	N/A	F	28	11,2	1985,6	1983,9	−1,7	N/A	N/A	N/A	research	No	coronal enamel
Analysis of 14 C and 13 C in teeth provides precise birth dating and clues to geographical origin	U7-26	Uruguay	N/A	F	26	3	1995,2	1993,1	−2,1	N/A	N/A	N/A	research	No	coronal enamel
Analysis of 14 C and 13 C in teeth provides precise birth dating and clues to geographical origin	U8-36	Uruguay	N/A	F	36	2,3	1992,3	1993,4	−1,1	N/A	N/A	N/A	research	No	coronal enamel
Analysis of 14 C and 13 C in teeth provides precise birth dating and clues to geographical origin	FM20	Irak	N/A	M	27	6,5	1975,8	1974,4	−1,4	N/A	N/A	N/A	research	No	coronal enamel
Analysis of 14 C and 13 C in teeth provides precise birth dating and clues to geographical origin	FM18	Quwait	N/A	M	48	13	1970,4	1968	−2,4	N/A	N/A	N/A	research	No	coronal enamel
Analysis of 14 C and 13 C in teeth provides precise birth dating and clues to geographical origin	FM19	N/A	N/A	M	28	12,6	1958,3	1960,8	−2,5	N/A	N/A	N/A	research	No	coronal enamel
Analysis of 14 C and 13 C in teeth provides precise birth dating and clues to geographical origin	FM19	N/A	N/A	M	38	13	1958,3	1957	−1,3	N/A	N/A	N/A	research	No	coronal enamel
Analysis of 14 C and 13 C in teeth provides precise birth dating and clues to geographical origin	FM21	N/A	N/A	F	28	11,2	1951,1	1986	−0,9	N/A	N/A	N/A	research	No	coronal enamel
Analysis of 14 C and 13 C in teeth provides precise birth dating and clues to geographical origin	FM58	N/A	N/A	M	21	3,3	1962,6	1959,8	−2,8	N/A	N/A	N/A	research	No	coronal enamel
Analysis of 14 C and 13 C in teeth provides precise birth dating and clues to geographical origin	FM04	N/A	N/A	F	14	4,9	1991,5	1991,5	−2,1	N/A	N/A	N/A	research	No	coronal enamel
Analysis of 14 C and 13 C in teeth provides precise birth dating and clues to geographical origin	FM13	N/A	N/A	M	24	5,6	1992,1	1992,1	−2,4	N/A	N/A	N/A	research	No	coronal enamel
Analysis of 14 C and 13 C in teeth provides precise birth dating and clues to geographical origin	FM14	N/A	N/A	F	17	5,8	1972,2	1972,2	0	N/A	N/A	N/A	research	No	coronal enamel
Analysis of 14 C and 13 C in teeth provides precise birth dating and clues to geographical origin	FM38	N/A	N/A	F	15	5,6	1989,1	1987,8	−1,3	N/A	N/A	N/A	research	No	coronal enamel
Analysis of 14 C and 13 C in teeth provides precise birth dating and clues to geographical origin	FM89	N/A	N/A	M	24	5,6	1947,3	Pre-Bomb	N/A	N/A	N/A	N/A	research	No	coronal enamel
Analysis of 14 C and 13 C in teeth provides precise birth dating and clues to geographical origin	FM89	N/A	N/A	M	25	6,6	1947,4	Pre-Bomb	N/A	N/A	N/A	N/A	research	No	coronal enamel
Analysis of 14 C and 13 C in teeth provides precise birth dating and clues to geographical origin	OC50	N/A	N/A	F	34	4,4	1912,8	Pre-Bomb	N/A	N/A	N/A	N/A	research	No	coronal enamel
Analysis of 14 C and 13 C in teeth provides precise birth dating and clues to geographical origin	OC54	N/A	N/A	F	34	4,4	1949	Pre-Bomb	N/A	N/A	N/A	N/A	research	No	coronal enamel
Analysis of 14 C and 13 C in teeth provides precise birth dating and clues to geographical origin	OC79	N/A	N/A	F	25	5,6	1937	Pre-Bomb	N/A	N/A	N/A	N/A	research	No	coronal enamel
Analysis of 14 C and 13 C in teeth provides precise birth dating and clues to geographical origin	J3	Japan	N/A	F	24	4,9	1950,3	Pre-Bomb	N/A	N/A	N/A	N/A	research	No	coronal enamel
Analysis of 14 C and 13 C in teeth provides precise birth dating and clues to geographical origin	J4	Japan	N/A	M	11	3,2	1932,3	Pre-Bomb	N/A	N/A	N/A	N/A	research	No	coronal enamel
Analysis of 14 C and 13 C in teeth provides precise birth dating and clues to geographical origin	J5	Japan	N/A	M	16	3,3	1928,2	Pre-Bomb	N/A	N/A	N/A	N/A	research	No	coronal enamel
Analysis of 14 C and 13 C in teeth provides precise birth dating and clues to geographical origin	J6	Japan	N/A	M	47	6,5	1912,7	Pre-Bomb	N/A	N/A	N/A	N/A	research	No	coronal enamel
Analysis of 14 C and 13 C in teeth provides precise birth dating and clues to geographical origin	J7	Japan	N/A	M	12	4	1917,3	Pre-Bomb	N/A	N/A	N/A	N/A	research	No	coronal enamel
Analysis of 14 C and 13 C in teeth provides precise birth dating and clues to geographical origin	J8	Japan	N/A	F	15	5,6	1911,3	Pre-Bomb	N/A	N/A	N/A	N/A	research	No	coronal enamel
Analysis of 14 C and 13 C in teeth provides precise birth dating and clues to geographical origin	J9	Japan	N/A	M	11	3,2	1917,4	Pre-Bomb	N/A	N/A	N/A	N/A	research	No	coronal enamel
Analysis of 14 C and 13 C in teeth provides precise birth dating and clues to geographical origin	J10	Japan	N/A	M	23	4,7	1931,4	N/A	N/A	N/A	N/A	N/A	research	No	coronal enamel
Analysis of 14 C and 13 C in teeth provides precise birth dating and clues to geographical origin	U1-42	Uruguay	N/A	M	42	3	N/A	Pre-Bomb	N/A	N/A	N/A	N/A	research	No	coronal enamel
Analysis of 14 C and 13 C in teeth provides precise birth dating and clues to geographical origin	U1-43	Uruguay	N/A	N/A	43	4,3	N/A	Pre-Bomb	N/A	N/A	N/A	N/A	research	No	coronal enamel
Analysis of 14 C and 13 C in teeth provides precise birth dating and clues to geographical origin	U4-27	Uruguay	N/A	M	27	6,5	1946,5	Pre-Bomb	N/A	N/A	N/A	N/A	research	No	coronal enamel
Analysis of 14 C and 13 C in teeth provides precise birth dating and clues to geographical origin	U5-12	Uruguay	N/A	M	12	4	1946,5	Pre-Bomb	N/A	N/A	N/A	N/A	research	No	coronal enamel
Analysis of 14 C and 13 C in teeth provides precise birth dating and clues to geographical origin	OC31	N/A	N/A	M	17	6,5	1952,3	1949,7	−2,6	N/A	N/A	N/A	research	No	coronal enamel
Analysis of 14 C and 13 C in teeth provides precise birth dating and clues to geographical origin	OC33	N/A	N/A	M	32	3	1953,8	Pre-Bomb	N/A	N/A	N/A	N/A	research	No	coronal enamel
Analysis of 14 C and 13 C in teeth provides precise birth dating and clues to geographical origin	OC34	N/A	N/A	M	33	4,2	1957,3	1957,3	0	N/A	N/A	N/A	research	No	coronal enamel
Analysis of 14 C and 13 C in teeth provides precise birth dating and clues to geographical origin	OC44	N/A	N/A	M	27	6,5	1952,7	1950,6	−2,1	N/A	N/A	N/A	research	No	coronal enamel
Analysis of 14 C and 13 C in teeth provides precise birth dating and clues to geographical origin	OC45	N/A	N/A	M	31	2,5	1958,2	1957,9	−0,3	N/A	N/A	N/A	research	No	coronal enamel
Analysis of 14 C and 13 C in teeth provides precise birth dating and clues to geographical origin	OC53	N/A	N/A	M	12	4	1952,1	Pre-Bomb	N/A	N/A	N/A	N/A	research	No	coronal enamel
Analysis of 14 C and 13 C in teeth provides precise birth dating and clues to geographical origin	OC56	N/A	N/A	M	36	2,4	1955,8	1952,7	−3,1	N/A	N/A	N/A	research	No	coronal enamel
Analysis of 14 C and 13 C in teeth provides precise birth dating and clues to geographical origin	OC58	N/A	N/A	M	26	3,3	1956,5	1953,9	−2,6	N/A	N/A	N/A	research	No	coronal enamel
Analysis of 14 C and 13 C in teeth provides precise birth dating and clues to geographical origin	OC60	N/A	N/A	F	17	5,8	1955,8	1954,1	−1,6	N/A	N/A	N/A	research	No	coronal enamel
Analysis of 14 C and 13 C in teeth provides precise birth dating and clues to geographical origin	OC66	N/A	N/A	M	28	12,6	1959,4	1949,8	−0,4	N/A	N/A	N/A	research	No	coronal enamel
Analysis of 14 C and 13 C in teeth provides precise birth dating and clues to geographical origin	FM19	Morocco	N/A	M	31	2,5	1958,3	1954	−4,3	N/A	N/A	N/A	research	No	coronal enamel
Analysis of 14 C and 13 C in teeth provides precise birth dating and clues to geographical origin	FM19	Morocco	N/A	M	32	3	N/A	1954,1	−4,2	N/A	N/A	N/A	research	No	coronal enamel
Application and implications of radiocarbon dating in forensic case work: when medico-legal significance meets archaeological relevance	22–182 C	France	48–58	F	43	1970–1972	N/A	1960–1964	N/A	N/A	N/A	N/A	forensic	No	enamel
Application and implications of radiocarbon dating in forensic case work: when medico-legal significance meets archaeological relevance	22-182D	France	48–58	F	13	1970–1972	N/A	1960–1964	N/A	N/A	N/A	N/A	forensic	No	enamel
BOMB PEAK: RADIOCARBON DATING OF SKELETAL REMAINS IN ROUTINE FORENSIC MEDICAL PRACTICE	G2	CZECH	55–59	M	first premolar	1970–1972	1957	1957	N/A	50–51	N/A	2013–2014	forensic	No	dentin
DETERMINING 14 C CONTENT IN DIFFERENT HUMAN TISSUES: IMPLICATIONS FOR APPLICATION OF 14 C BOMB-SPIKE DATING IN FORENSIC MEDICINE	S2	italy	36	M	mandibular right canine	4,5 +/- 1,5	1973	1971,5+/−2,2	N/A	N/A	N/A	2009,8 +/- 0,2	forensic	No	enamel
DETERMINING 14 C CONTENT IN DIFFERENT HUMAN TISSUES: IMPLICATIONS FOR APPLICATION OF 14 C BOMB-SPIKE DATING IN FORENSIC MEDICINE	S3	italy	36	M	mandibular right second molar	5,5 +/- 2,5	1973	1973,1+/−3,2	N/A	N/A	N/A	2009,8 +/- 0,3	forensic	No	enamel
IMPROVED APPLICATION OF BOMB CARBON IN TEETH FOR FORENSIC INVESTIGATION	MT-1	CHINA	N/A	M	Second Molar	1975 +/−0,7	1968	N/A	−0,1	N/A	N/A	N/A	research	No	enamel
IMPROVED APPLICATION OF BOMB CARBON IN TEETH FOR FORENSIC INVESTIGATION	MT-2	CHINA	N/A	M	Tird Molar	1984,8 +/−1	1968	N/A	2,8	N/A	N/A	N/A	research	No	enamel
IMPROVED APPLICATION OF BOMB CARBON IN TEETH FOR FORENSIC INVESTIGATION	MT-3	CHINA	N/A	M	Tird Molar	2002 +/−1	1983	N/A	5	N/A	N/A	N/A	research	No	enamel
IMPROVED APPLICATION OF BOMB CARBON IN TEETH FOR FORENSIC INVESTIGATION	MT-4	CHINA	N/A	M	Tird Molar	1996,5 +/- 0,9	1983	N/A	−0,5	N/A	N/A	N/A	research	No	enamel
IMPROVED APPLICATION OF BOMB CARBON IN TEETH FOR FORENSIC INVESTIGATION	MT-5	CHINA	N/A	M	First Molar	1978,8 +/- 0,9	1978	N/A	−2	N/A	N/A	N/A	research	No	enamel
IMPROVED APPLICATION OF BOMB CARBON IN TEETH FOR FORENSIC INVESTIGATION	MT-6	CHINA	N/A	M	First Molar	1996,7 +/−0,7	1980	N/A	2,7	N/A	N/A	N/A	research	No	enamel
IMPROVED APPLICATION OF BOMB CARBON IN TEETH FOR FORENSIC INVESTIGATION	MT-7	CHINA	N/A	F	Tird Molar	1994,5 +/- 1,6	1980	N/A	0,5	N/A	N/A	N/A	research	No	enamel
IMPROVED APPLICATION OF BOMB CARBON IN TEETH FOR FORENSIC INVESTIGATION	MT-8	CHINA	N/A	M	Tird Molar	1980,3 +/- 0,5	1968	N/A	−1,7	N/A	N/A	N/A	research	No	enamel
IMPROVED APPLICATION OF BOMB CARBON IN TEETH FOR FORENSIC INVESTIGATION	MT-9	CHINA	N/A	M	Tird Molar	1981 +/- 1	1968	N/A	1	N/A	N/A	N/A	research	No	enamel
IMPROVED APPLICATION OF BOMB CARBON IN TEETH FOR FORENSIC INVESTIGATION	TB-06	CHINA	N/A	F	First Molar	1932 +/- 14	1930	N/A	0,8	N/A	N/A	N/A	research	No	enamel
IMPROVED APPLICATION OF BOMB CARBON IN TEETH FOR FORENSIC INVESTIGATION	TB-28	CHINA	N/A	F	Second Molar	1936.8 ± 19.0	1952	N/A	–22.7	N/A	N/A	N/A	research	No	enamel
IMPROVED APPLICATION OF BOMB CARBON IN TEETH FOR FORENSIC INVESTIGATION	TB-54	CHINA	N/A	M	Tird Molar	1961.6 ± 0.3	1966	N/A	–2.2	N/A	N/A	N/A	research	No	enamel
A PRELIMINARY ASSESSMENT OF AGE AT DEATH DETERMINATION USING THE NUCLEAR WEAPONS TESTING 14 C ACTIVITY OF DENTINE AND ENAMEL	N/A	northern hemisphere	N/A	M	wisdom tooth	1960–1961 o 1980–1982	1969	1969–1971	0	N/A	N/A	N/A	research	No	enamel
A PRELIMINARY ASSESSMENT OF AGE AT DEATH DETERMINATION USING THE NUCLEAR WEAPONS TESTING 14 C ACTIVITY OF DENTINE AND ENAMEL	N/A	northern hemisphere	N/A	M	lower left 1	1961–1962 o 1975–1977	1961	1973–1975	0–2	N/A	N/A	N/A	research	No	enamel
A PRELIMINARY ASSESSMENT OF AGE AT DEATH DETERMINATION USING THE NUCLEAR WEAPONS TESTING 14 C ACTIVITY OF DENTINE AND ENAMEL	N/A	northern hemisphere	N/A	M	upper right 7	1962 o 1975–1976	1971	1970–1971	12	N/A	N/A	N/A	research	No	enamel
A PRELIMINARY ASSESSMENT OF AGE AT DEATH DETERMINATION USING THE NUCLEAR WEAPONS TESTING 14 C ACTIVITY OF DENTINE AND ENAMEL	N/A	northern hemisphere	N/A	M	lower left 7	1960–1961 o 1982–1984	1979	1976–1979	0	N/A	N/A	N/A	research	No	enamel
A PRELIMINARY ASSESSMENT OF AGE AT DEATH DETERMINATION USING THE NUCLEAR WEAPONS TESTING 14 C ACTIVITY OF DENTINE AND ENAMEL	N/A	northern hemisphere	N/A	M	lower left 6	1958–1961 o 1983–1985	1983	1982–1984	0	N/A	N/A	N/A	research	No	enamel
A PRELIMINARY ASSESSMENT OF AGE AT DEATH DETERMINATION USING THE NUCLEAR WEAPONS TESTING 14 C ACTIVITY OF DENTINE AND ENAMEL	N/A	northern hemisphere	N/A	M	upper right 4	1957 o 1991–1994	1989	1988–1990	0	N/A	N/A	N/A	research	No	enamel
A PRELIMINARY ASSESSMENT OF AGE AT DEATH DETERMINATION USING THE NUCLEAR WEAPONS TESTING 14 C ACTIVITY OF DENTINE AND ENAMEL	N/A	northern hemisphere	N/A	M	lower right 4	1952–1954 o 1998–2002	2000	1994–1999	0	N/A	N/A	N/A	research	No	enamel
Analysis of Radiocarbon, Stable Isotopes and DNA in Teeth to Facilitate Identification of Unknown Decedents	1	USA	N/A	M	41	2,5	1965,3	1963,1	−2,2	1,541	0,0067	N/A	research	No	enamel
Analysis of Radiocarbon, Stable Isotopes and DNA in Teeth to Facilitate Identification of Unknown Decedents	2	USA	N/A	F	42	2,8	1918,4	pre-bomb	pre bomb	0,9986	0,0038	N/A	research	No	enamel
Analysis of Radiocarbon, Stable Isotopes and DNA in Teeth to Facilitate Identification of Unknown Decedents	3	USA	N/A	M	15	6,6	1974	1970,1	−3,9	1,5361	0,0045	N/A	research	No	enamel
Analysis of Radiocarbon, Stable Isotopes and DNA in Teeth to Facilitate Identification of Unknown Decedents	4	USA	N/A	M	44	5,1	2001,7	1996,3	−5,4	1,113	0,0042	N/A	research	No	enamel
Analysis of Radiocarbon, Stable Isotopes and DNA in Teeth to Facilitate Identification of Unknown Decedents	5	USA	N/A	F	36	2,3	1972,6	1967,4	3,8	1,3645	0,0051	N/A	research	No	enamel
Analysis of Radiocarbon, Stable Isotopes and DNA in Teeth to Facilitate Identification of Unknown Decedents	6	USA	N/A	M	22	4	1966,2	1966,8	0,7	1,6698	0,0072	N/A	research	No	enamel
Analysis of Radiocarbon, Stable Isotopes and DNA in Teeth to Facilitate Identification of Unknown Decedents	7	USA	N/A	M	38	13	1995,3	1992,5	−2,8	1,1357	0,004	N/A	research	No	enamel
Analysis of Radiocarbon, Stable Isotopes and DNA in Teeth to Facilitate Identification of Unknown Decedents	7	USA	N/A	M	48	13	1995,3	1993,1	−2,2	1,1323	0,0042	N/A	research	No	enamel
Analysis of Radiocarbon, Stable Isotopes and DNA in Teeth to Facilitate Identification of Unknown Decedents	8	USA	N/A	F	27	5,8	1968,6	1967,2	−1,4	1,6459	0,0059	N/A	research	No	enamel
Analysis of Radiocarbon, Stable Isotopes and DNA in Teeth to Facilitate Identification of Unknown Decedents	8	USA	N/A	F	45	5,7	1968,5	1967,4	−1,1	1,6356	0,0057	N/A	research	No	enamel
Analysis of Radiocarbon, Stable Isotopes and DNA in Teeth to Facilitate Identification of Unknown Decedents	9	USA	N/A	M	17	6,5	1989,8	1987,5	−2,3	1,182	0,0042	N/A	research	No	enamel
Analysis of Radiocarbon, Stable Isotopes and DNA in Teeth to Facilitate Identification of Unknown Decedents	10	USA	N/A	M	47	6,5	1979,9	1977,5	−2,4	1,3421	0,0047	N/A	research	No	enamel
Analysis of Radiocarbon, Stable Isotopes and DNA in Teeth to Facilitate Identification of Unknown Decedents	11	USA	N/A	F	24	4,9	1964,9	1962,4	−2,5	1,3585	0,005	N/A	research	No	enamel
Analysis of Radiocarbon, Stable Isotopes and DNA in Teeth to Facilitate Identification of Unknown Decedents	12	USA	N/A	M	16	3,3	1973,9	1971,8	−2,1	1,4898	0,0053	N/A	research	No	enamel
Analysis of Radiocarbon, Stable Isotopes and DNA in Teeth to Facilitate Identification of Unknown Decedents	12	USA	N/A	M	37	6,5	1977,1	1976,8	−0,3	1,3518	0,0048	N/A	research	No	enamel
Analysis of Radiocarbon, Stable Isotopes and DNA in Teeth to Facilitate Identification of Unknown Decedents	13	USA	N/A	M	15	6,6	1998,4	1995,6	−2,8	1,117	0,004	N/A	research	No	enamel
Analysis of Radiocarbon, Stable Isotopes and DNA in Teeth to Facilitate Identification of Unknown Decedents	14	USA	N/A	M	17	6,5	1991	1989,4	−1,6	1,1646	0,0041	N/A	research	No	enamel
Analysis of Radiocarbon, Stable Isotopes and DNA in Teeth to Facilitate Identification of Unknown Decedents	14	USA	N/A	M	18	12,6	1997,1	1994,3	−2,8	1,1242	0,006	N/A	research	No	enamel
Analysis of Radiocarbon, Stable Isotopes and DNA in Teeth to Facilitate Identification of Unknown Decedents	14	USA	N/A	M	28	12,6	1997,1	1996,5	−0,6	1,1118	0,0039	N/A	research	No	enamel
Analysis of Radiocarbon, Stable Isotopes and DNA in Teeth to Facilitate Identification of Unknown Decedents	15	USA	N/A	F	25	5,6	1975,4	1975,8	0,4	1,3675	0,0048	N/A	research	No	enamel
Analysis of Radiocarbon, Stable Isotopes and DNA in Teeth to Facilitate Identification of Unknown Decedents	15	USA	N/A	F	26	3	1972,8	1972,2	−0,6	1,475	0,0052	N/A	research	No	enamel
Analysis of Radiocarbon, Stable Isotopes and DNA in Teeth to Facilitate Identification of Unknown Decedents	16	UK/USA	N/A	F	26	3	1990,6	1988	−2,6	1,1765	0,0046	N/A	research	No	enamel
Analysis of Radiocarbon, Stable Isotopes and DNA in Teeth to Facilitate Identification of Unknown Decedents	17	USA	N/A	M	45	6,5	1964,2	1962	−1,7	1,2773	0,0036	N/A	research	No	enamel
Analysis of Radiocarbon, Stable Isotopes and DNA in Teeth to Facilitate Identification of Unknown Decedents	18	USA	N/A	M	44	5,1	2002,3	2000	−2,3	1,094	0,0039	N/A	research	No	enamel
Analysis of Radiocarbon, Stable Isotopes and DNA in Teeth to Facilitate Identification of Unknown Decedents	18	USA	N/A	M	14	5,6	2002,8	2000	−2,8	1,0922	0,0031	N/A	research	No	enamel
Analysis of Radiocarbon, Stable Isotopes and DNA in Teeth to Facilitate Identification of Unknown Decedents	19	USA	N/A	M	14	5,6	2001,9	1999,2	−2,7	1,0971	0,0038	N/A	research	No	enamel
Analysis of Radiocarbon, Stable Isotopes and DNA in Teeth to Facilitate Identification of Unknown Decedents	19	USA	N/A	M	34	5,1	2001,4	2001	−0,4	1,0872	0,0038	N/A	research	No	enamel
Analysis of Radiocarbon, Stable Isotopes and DNA in Teeth to Facilitate Identification of Unknown Decedents	20	USA	N/A	F	31	2,5	1947,4	Pre-Bomb	pre- bomb	1,0134	0,0036	N/A	research	No	enamel
Analysis of Radiocarbon, Stable Isotopes and DNA in Teeth to Facilitate Identification of Unknown Decedents	20	USA	N/A	F	33	4,1	1949	Pre-Bomb	pre bomb	0,992	0,0029	N/A	research	No	enamel
Analysis of Radiocarbon, Stable Isotopes and DNA in Teeth to Facilitate Identification of Unknown Decedents	21	USA	N/A	M	15	6,6	1959,7	1957,3	−2,4	1,0496	0,0044	N/A	research	No	enamel
Analysis of Radiocarbon, Stable Isotopes and DNA in Teeth to Facilitate Identification of Unknown Decedents	22	USA	N/A	M	28	12,6	1992,8	1991,6	−1,2	1,1462	0,004	N/A	research	No	enamel
Analysis of Radiocarbon, Stable Isotopes and DNA in Teeth to Facilitate Identification of Unknown Decedents	23	USA	N/A	M	42	3	1984,1	1983,3	−0,7	1,2281	0,0038	N/A	research	No	enamel
Analysis of Radiocarbon, Stable Isotopes and DNA in Teeth to Facilitate Identification of Unknown Decedents	24	USA	N/A	F	23	4,7	1958,1	1956,1	−2	1,0171	0,0036	N/A	research	No	enamel
Analysis of Radiocarbon, Stable Isotopes and DNA in Teeth to Facilitate Identification of Unknown Decedents	25	USA	N/A	F	14	4,9	1996,3	1992,1	−4,2	1,1396	0,004	N/A	research	No	enamel
Analysis of Radiocarbon, Stable Isotopes and DNA in Teeth to Facilitate Identification of Unknown Decedents	25	USA	N/A	F	34	4,4	1995,8	1994,6	−1,2	1,1224	0,0032	N/A	research	No	enamel
Analysis of Radiocarbon, Stable Isotopes and DNA in Teeth to Facilitate Identification of Unknown Decedents	26	USA	N/A	M	14	5,6	1945,9	Pre-bomb	Pre-bomb	0,9948	0,0028	N/A	research	No	enamel
Analysis of Radiocarbon, Stable Isotopes and DNA in Teeth to Facilitate Identification of Unknown Decedents	26	USA	N/A	M	31	2,5	1942,8	Pre-bomb	Pre-bomb	0,99	0,0032	N/A	research	No	enamel
Analysis of Radiocarbon, Stable Isotopes and DNA in Teeth to Facilitate Identification of Unknown Decedents	27	USA	N/A	M	23	4,7	1960,8	1958,8	−2	1,1726	0,0036	N/A	research	No	enamel
Analysis of Radiocarbon, Stable Isotopes and DNA in Teeth to Facilitate Identification of Unknown Decedents	28	USA	N/A	M	36	2,4	1994,4	1998,2	3,8	1,1019	0,0035	N/A	research	No	enamel
Analysis of Radiocarbon, Stable Isotopes and DNA in Teeth to Facilitate Identification of Unknown Decedents	29	USA	N/A	M	18	12,6	1976,5	1975,4	−2,5	1,3799	0,0038	N/A	research	No	enamel
Analysis of Radiocarbon, Stable Isotopes and DNA in Teeth to Facilitate Identification of Unknown Decedents	29	USA	N/A	M	46	2,4	1967,6	1962,7	−5	1,4075	0,0041	N/A	research	No	enamel
Analysis of Radiocarbon, Stable Isotopes and DNA in Teeth to Facilitate Identification of Unknown Decedents	30	USA	N/A	M	12	4	1964,1	1962,1	2	1,3087	0,0038	N/A	research	No	enamel
Analysis of Radiocarbon, Stable Isotopes and DNA in Teeth to Facilitate Identification of Unknown Decedents	30	USA	N/A	M	13	4,7	1964,8	1962,4	−2,4	1,3502	0,0041	N/A	research	No	enamel
Analysis of Radiocarbon, Stable Isotopes and DNA in Teeth to Facilitate Identification of Unknown Decedents	30	USA	N/A	M	14	5,6	1965,7	1962,9	−2,8	1,4744	0,0062	N/A	research	No	enamel
Analysis of Radiocarbon, Stable Isotopes and DNA in Teeth to Facilitate Identification of Unknown Decedents	30	USA	N/A	M	22	4	1964,1	1963,3	−0,8	1,6172	0,0066	N/A	research	No	enamel
Analysis of Radiocarbon, Stable Isotopes and DNA in Teeth to Facilitate Identification of Unknown Decedents	30	USA	N/A	M	23	4,7	1964,8	1965,8	−1	1,7399	0,0267	N/A	research	No	enamel
Analysis of Radiocarbon, Stable Isotopes and DNA in Teeth to Facilitate Identification of Unknown Decedents	30	USA	N/A	M	26	3,3	1963,4	1961,1	−2,3	1,2254	0,0046	N/A	research	No	enamel
Analysis of Radiocarbon, Stable Isotopes and DNA in Teeth to Facilitate Identification of Unknown Decedents	30	USA	N/A	M	31	2,5	1962,6	1962	−0,6	1,2791	0,0073	N/A	research	No	enamel
Analysis of Radiocarbon, Stable Isotopes and DNA in Teeth to Facilitate Identification of Unknown Decedents	30	USA	N/A	M	32	3	1963,1	1963,3	0,2	1,6571	0,1693	N/A	research	No	enamel
Analysis of Radiocarbon, Stable Isotopes and DNA in Teeth to Facilitate Identification of Unknown Decedents	30	USA	N/A	M	37	6,5	1966,6	1966,8	0,2	1,6735	0,0056	N/A	research	No	enamel
Analysis of Radiocarbon, Stable Isotopes and DNA in Teeth to Facilitate Identification of Unknown Decedents	30	USA	N/A	M	41	2,5	1962,6	1962,6	0	1,4131	0,0059	N/A	research	No	enamel
Analysis of Radiocarbon, Stable Isotopes and DNA in Teeth to Facilitate Identification of Unknown Decedents	30	USA	N/A	M	42	3	1963,1	1962,8	−0,3	1,5051	0,1504	N/A	research	No	enamel
Analysis of Radiocarbon, Stable Isotopes and DNA in Teeth to Facilitate Identification of Unknown Decedents	30	USA	N/A	M	43	4,3	1964,4	1962,8	−1,6	1,4475	0,0062	N/A	research	No	enamel
Analysis of Radiocarbon, Stable Isotopes and DNA in Teeth to Facilitate Identification of Unknown Decedents	30	USA	N/A	M	44	5,1	1965,2	1966,4	1,2	1,6666	0,0067	N/A	research	No	enamel
Analysis of Radiocarbon, Stable Isotopes and DNA in Teeth to Facilitate Identification of Unknown Decedents	30	USA	N/A	M	47	6,5	1966,6	1966,5	−0,1	1,6945	0,006	N/A	research	No	enamel
Analysis of Radiocarbon, Stable Isotopes and DNA in Teeth to Facilitate Identification of Unknown Decedents	31	USA	N/A	M	11	3,2	1979,6	1977	−2,6	1,35	0,0048	N/A	research	No	enamel
Analysis of Radiocarbon, Stable Isotopes and DNA in Teeth to Facilitate Identification of Unknown Decedents	32	Mexico	N/A	F	46	2,4	1946,9	Pre-Bomb	Pre-bomb	0,9849	0,0035	N/A	research	No	enamel
Analysis of Radiocarbon, Stable Isotopes and DNA in Teeth to Facilitate Identification of Unknown Decedents	33	Mexico	N/A	M	23	4,7	1956,8	1956	−0,8	1,028	0,004	N/A	research	No	enamel
Analysis of Radiocarbon, Stable Isotopes and DNA in Teeth to Facilitate Identification of Unknown Decedents	34	Mexico	N/A	M	26	3,3	1962,8	1960	−2,8	1,2163	0,0045	N/A	research	No	enamel
Analysis of Radiocarbon, Stable Isotopes and DNA in Teeth to Facilitate Identification of Unknown Decedents	35	Mexico	N/A	F	46	2,3	1945,7	Pre-Bomb	Pre-Bomb	0,9945	0,0035	N/A	research	No	enamel
Analysis of Radiocarbon, Stable Isotopes and DNA in Teeth to Facilitate Identification of Unknown Decedents	36	Mexico	N/A	M	37	6,5	1961,3	1962,7	1,4	1,6573	0,0059	N/A	research	No	enamel
Analysis of Radiocarbon, Stable Isotopes and DNA in Teeth to Facilitate Identification of Unknown Decedents	36	Mexico	N/A	M	36	2,4	1957,2	1957,4	0,2	1,0738	0,0038	N/A	research	No	enamel
Analysis of Radiocarbon, Stable Isotopes and DNA in Teeth to Facilitate Identification of Unknown Decedents	37	Mexico	N/A	F	35	5,7	1926,7	Pre-Bomb	Pre-Bomb	0,992	0,0033	N/A	research	No	enamel
Analysis of Radiocarbon, Stable Isotopes and DNA in Teeth to Facilitate Identification of Unknown Decedents	38	Mexico	N/A	F	36	2,3	1933,5	Pre-Bomb	Pre-Bomb	0,9949	0,0034	N/A	research	No	enamel
Analysis of Radiocarbon, Stable Isotopes and DNA in Teeth to Facilitate Identification of Unknown Decedents	39	Mexico	N/A	F	17	5,8	1969,6	1970,1	0,5	1,5306	0,0054	N/A	research	No	enamel
Analysis of Radiocarbon, Stable Isotopes and DNA in Teeth to Facilitate Identification of Unknown Decedents	39	Mexico	N/A	F	45	5,7	1969,5	1970,2	0,7	1,5331	0,0058	N/A	research	No	enamel
RADIOCARBON ANALYSIS OF DENTAL ENAMEL AND BONE TO EVALUATE DATE OF BIRTH AND DEATH: PERSPECTIVE FROM THE SOUTHERN HEMISPHERE	A-1	Peru	44	N/A	46	1948–1951	1947	before Bomb-curve	N/A	N/A	N/A	1991	research/forensic	No	enamel
RADIOCARBON ANALYSIS OF DENTAL ENAMEL AND BONE TO EVALUATE DATE OF BIRTH AND DEATH: PERSPECTIVE FROM THE SOUTHERN HEMISPHERE	B-1	Peru	56	N/A	17	1944–1948	1941	before Bomb-curve	N/A	N/A	N/A	1997	research/forensic	No	enamel
RADIOCARBON ANALYSIS OF DENTAL ENAMEL AND BONE TO EVALUATE DATE OF BIRTH AND DEATH: PERSPECTIVE FROM THE SOUTHERN HEMISPHERE	C-1	Peru	27	N/A	27	1967–1971	1964	1965–1967	−2	N/A	N/A	1991	research/forensic	No	enamel
RADIOCARBON ANALYSIS OF DENTAL ENAMEL AND BONE TO EVALUATE DATE OF BIRTH AND DEATH: PERSPECTIVE FROM THE SOUTHERN HEMISPHERE	D-1	Peru	16	N/A	46	1968–1971	1968	1964;1968–1970	0	N/A	N/A	1984	research/forensic	No	enamel

## Results

### Study selection

The study selection process followed the PRISMA 2020 guidelines to ensure transparency and reproducibility. A total of 5,918 records were initially identified from three major databases: PubMed (*n* = 318), Scopus (*n* = 907), and Web of Science (*n* = 4,693). After removing 633 duplicate entries, the remaining 5,285 records were screened based on titles and abstracts. Of these, 5,279 were excluded owing to irrelevance to the inclusion criteria, leaving six full-text reports considered for further evaluation. However, three of these reports could not be retrieved in full, resulting in three studies being assessed for eligibility.

In addition to the database searches, six additional records were identified through citation tracking, with no studies found on websites or in other sources.

**Fig. 1:**
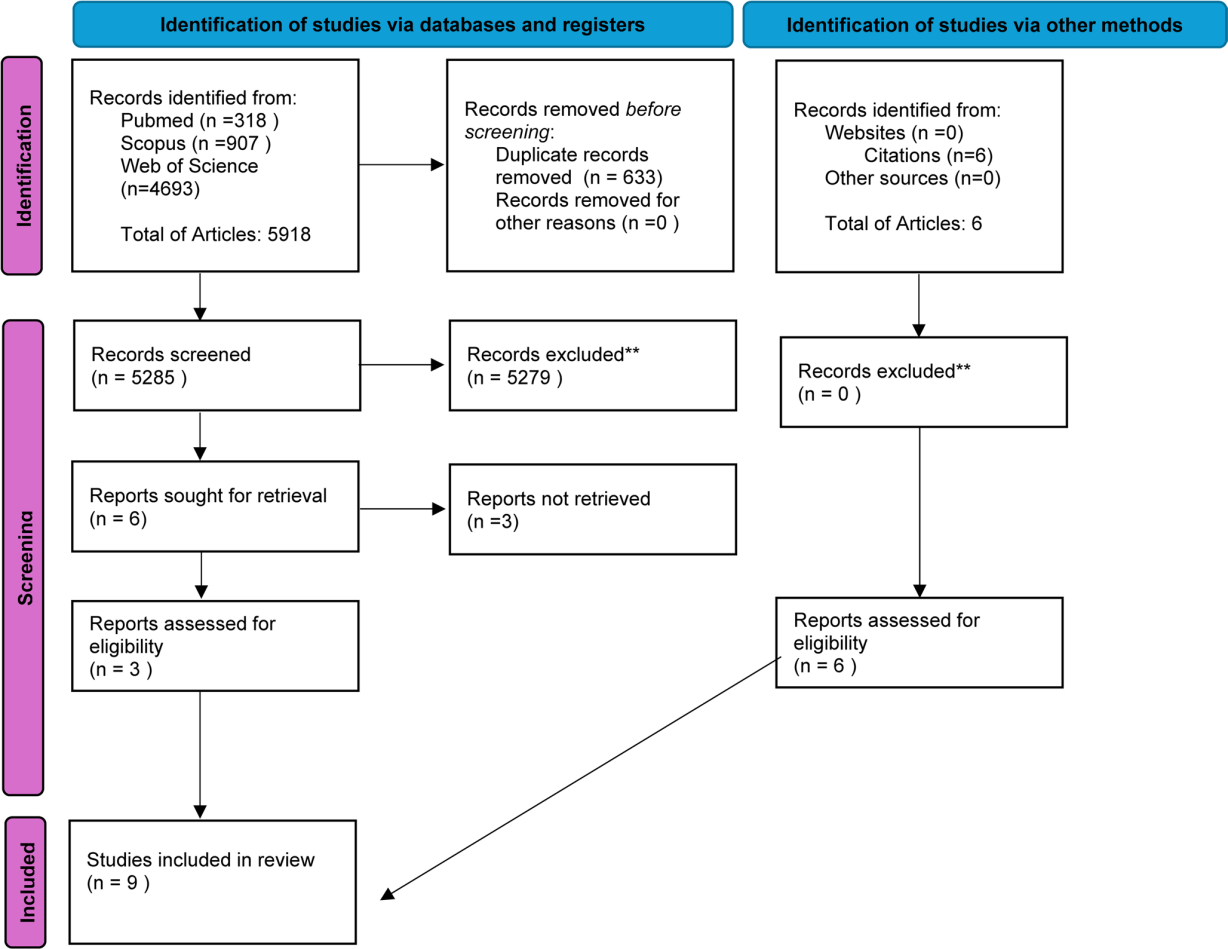
PRISMA 2020 flow diagram for new systematic reviews which included searches of databases and registers only

Ultimately, nine studies met the inclusion criteria and were included in the final review. This selection process is visually represented in the PRISMA flow diagram (Fig. 1) and reflects a rigorous approach in identifying studies focused on radiocarbon dating of dental tissues for forensic and anthropological applications.

### Included studies

The nine included studies were published between 2006 and 2024, encompassing data from over 270 individual teeth. These studies involved analyses of dental tissues including enamel, dentin, and cementum—either separately or in combination—to estimate year of birth (YOB), year of death (YOD), or both.

In most investigations, accelerator mass spectrometry was used for radiocarbon analysis and calibration programs, such as CALIBomb, were used to correlate measured C-14 values with atmospheric bomb-pulse curves. Samples included molars, premolars, incisors, and canines, with a preference for the first and second molars owing to their early and well-documented development, as well as their larger size and higher volume of dental tissue, facilitating sample collection and improving analytical reliability [16].

### Dental tissues

Enamel yielded the most accurate results, with a mean absolute error (MAE) of ± 1.3–1.9 years referring to the precision of the radiocarbon-derived enamel formation year, as calibrated against the atmospheric bomb curve [15, 23, 25]. The year of birth (YOB) is subsequently obtained by subtracting the tooth-specific crown formation time from this radiocarbon-derived enamel date; therefore, the overall YOB uncertainty results from both the ¹⁴C analytical error and the biological variability in enamel development timing. Conversely, enamel formed before 1950 resulted in broader chronological estimates owing to the flatter baseline of atmospheric C-14 [16, 25]. The mean absolute error in YOB estimation post-1963 enamel highlights the strong correlation of the method with the atmospheric C-14 peak [15, 23]. In cases where both enamel and dentin were analyzed, enamel provided a more reliable estimate owing to its metabolic stability, whereas dentin occasionally helped narrow down the potential age range [24]. Ubelaker DH et al. (2011) demonstrated the utility of comparing enamel and bone C-14 values to determine both YOB and YOD when complete skeletal profiles are available [5].

The included studies showed that teeth, especially enamel, offer a reliable substrate for radiocarbon dating owing to their lack of post-formational remodeling. Once formed during early childhood, enamel becomes metabolically inert and does not undergo any turnover, thereby preserving the original C-14 signal incorporated during its formation. This stability is attributed to its acellular and avascular nature, lacking the capacity for regeneration or repair [26]. In contrast, dentin and cementum display distinct biological behaviors [27]. Dentin may undergo limited secondary and tertiary apposition throughout life, particularly in response to aging or injury, which can alter its carbon composition [28, 29]. Cementum, especially its cellular form, continues to accumulate in incremental layers through appositional growth, a process that persists throughout life and can incorporate more recent atmospheric carbon, potentially altering chronological resolution [30]. These differences in post-formational dynamics explain why enamel provides the most temporally stable matrix.

Alkass et al. and Calcagnile et al. successfully combined C-14 dating with stable isotope analyses (C-14 and O-18) and DNA typing to improve forensic identification strategies [13, 15]. Radiocarbon analysis of dental enamel was used to estimate YOB with high precision by comparing the measured C-14 levels with the atmospheric bomb curve [15]. Simultaneously, stable isotope ratios, particularly differences in C-13 composition, were analyzed to infer dietary patterns and geographic origin, based on regional and ecological differences in plant photosynthesis pathways (C3 vs. C4). For example, Alkass et al. showed that C-13 values in enamel varied significantly among individuals from Scandinavia, who had the lowest values, followed by those from Japan, the Middle East, and South America, thus offering a means to infer the regional background of the subjects [15].

## Discussion

Radiocarbon analysis of dental tissues offers a valuable tool for estimating YOB or YOD, primarily because of the biological stability of enamel and its ability to retain the atmospheric C-14 signature from the time of formation [13, 15]. Nevertheless, the data reviewed in the present study highlight several critical considerations that may affect the accuracy and reliability of dental radiocarbon analysis.

In practical forensic settings, analysis of isolated tissues (e.g., enamel vs. dentin) is often not feasible. Whole-tooth sampling, although less precise in terms of tissue-specific formation time, represents a realistic and efficient solution when dealing with compromised remains or minimal sample availability. Several studies have shown that even with whole-tooth analysis, deviations from known dates remain within acceptable forensic margins [15, 17, 31].

In the analyzed cases, enamel showed the highest chronological reliability, with radiocarbon-derived enamel formation dates differing from true values by an average of ± 1.3–1.9 years and rarely exceeding 3 years. These values represent the mean absolute error for enamel formation year estimation, not directly for year of birth (YOB), which carries an additional component of uncertainty due to biological variation in crown-formation timing. When converted into YOB estimates, discrepancies generally remained within a ± 2–3-year window, consistent with the results reported by some authors [23–25]. Nevertheless, as noted by Saitoh et al., the amount of usable enamel may be limited in practical forensic contexts owing to the requested enamel quantity being approximately 0.5–1 g [4, 16, 22]. In such cases, whole-tooth sampling, including dentin and cementum, becomes necessary. However, this approach introduces a potential temporal discrepancy between enamel formation and subsequent, more recently developed portions, such as dentin or cementum. In our dataset, the average lag time ranged from − 0.3 to over 3 years, with higher discrepancies observed when dentin was included. This reflects the ongoing appositional growth and metabolic activity of dentin and cementum, which may incorporate more recent atmospheric carbon over time [27, 30].

Another significant limitation of this approach involves geographic and dietary variability in atmospheric C-14 levels. While calibration curves, such as IntCal20 (northern hemisphere) and SHCal20 (southern hemisphere), account for regional differences, the lack of information on an individual’s origin complicates the choice of the correct calibration model [21, 32, 33]. Ancestry evaluations, which are fundamental for determining subject origin, rely only on morphological characterization. In most forensic cases, information regarding a sample’s origin or any other general information is lacking and insufficient to determine the precise geographic provenance, thus potentially impairing accurate chronological interpretation [3]. Future studies should focus on explicitly reporting the hemisphere of sample origin and selecting the appropriate calibration curve (e.g., IntCal20 vs. SHCal20) to ensure dating consistency.

Although dental tissues, particularly enamel, can be valuable for establishing year of birth and for identifying individuals in degraded or incomplete remains, they should not be considered as a first choice. Tissues characterized by shorter carbon turnover, such as hair, nail, or bones, offer a closer temporal relationship with atmospheric radiocarbon and thus more accurate time-of-death estimates, particularly in older adults, and in the case of older individuals whose death occurred in modern times, but whose teeth formed before 1950, in the pre-bomb phase, prior to the ascending phase of the curve [3].

## Conclusion

Radiocarbon dating of teeth is an asset in forensic identification owing to the temporal and environmental information preserved within dental tissues, especially enamel. However, practical limitations related to sample quantity, tissue selection, and inter-individual variability must be carefully considered when interpreting results.

Dental studies have often included limited sample sizes. Unlike large-scale studies on bone dating, this limitation prevents comprehensive statistical analysis, even when the data are grouped. Furthermore, in real forensic cases, separating individual dental tissues is almost always impractical; therefore, the tooth must be considered as a single element.

Geographic and dietary influences on C-14 distribution, combined with the challenge of accurately determining an individual’s region of origin (common in forensic cases), complicate the use of global calibration curves. These challenges reinforce the need for integrated methodologies that combine radiocarbon analysis with other forensic tools. These considerations underscore the importance of a multidisciplinary approach to the analysis of unidentified human remains. The integration of radiocarbon dating with stable isotope analysis (e.g., C-13 and O-18) and genetic testing can enhance the overall accuracy of biological profiling, as demonstrated by Alkass et al. and Thevissen et al. [6, 18].

In conclusion, although not without methodological constraints, dental radiocarbon analysis can be a useful tool for indirect PMI assessment and individual identification. This approach should be integrated into a multidisciplinary forensic framework to improve the accuracy and reliability of postmortem reconstructions. Teeth are a promising tool for dating, as YOB correlates with YOD and age—both of which are fundamental parameters in forensics. However, further studies involving larger samples and whole-tooth analyses are needed, without differentiating between enamel, dentin, and cementum, owing to the practical difficulty of diversifying tissues from the same tooth in sufficient, uncontaminated quantities. Although this implies a slightly longer interval (approximately 3 years), the results remain sufficient and useful for investigations.

## Data Availability

All data supporting the findings of this study are available in the published articles included in the systematic review and cited within the manuscript.
